# Landscape of rare‐allele variants in cultivated and wild soybean genomes

**DOI:** 10.1002/tpg2.70020

**Published:** 2025-03-27

**Authors:** Zhi Liu, Xiaolei Shi, Qing Yang, Ying Li, Chunyan Yang, Mengchen Zhang, Yong‐Qiang Charles An, Henry T. Nguyen, Long Yan, Qijian Song

**Affiliations:** ^1^ Hebei Key Laboratory of Crop Genetics and Breeding, National Soybean Improvement Center Shijiazhuang Sub‐Center, Huang‐Huai‐Hai Key Laboratory of Biology and Genetic Improvement of Soybean, Ministry of Agriculture and Rural Affairs, Institute of Cereal and Oil Crops Hebei Academy of Agricultural and Forestry Sciences Shijiazhuang China; ^2^ USDA‐ARS Midwest Area, Plant Genetics Research Unit St. Louis Missouri USA; ^3^ Donald Danforth Plant Science Center St. Louis Missouri USA; ^4^ Division of Plant Science and Technology University of Missouri Columbia Missouri USA; ^5^ USDA‐ARS Soybean Genomics and Improvement Laboratory Beltsville Maryland USA

## Abstract

Rare‐allele variants are important for crop improvement because they can be linked to important traits. However, genome‐wide distribution and annotation of rare‐allele variants have not been reported. We analyzed sequencing data from 1556 soybean accessions and found 6,533,419 rare‐allele variants in *Glycine max* and 941,274 in *Glycine soja* populations. Although the total number of variants was 20% less in *G. max* than *G. soja*, the number of rare‐allele variants in *G. max* was six times that in *G. soja*. Among the rare‐allele variants in *G. max*, 19.16% were novel mutations that did not exist in *G. soja*. Domestication and artificial selection have not only reduced overall genetic diversity but also the frequency of variants of cultivated soybean. Rare‐allele variants were mainly located in intergenic and noncoding regions rather than coding regions, and in heterochromatin regions rather than euchromatic regions. There were 121,450 rare‐allele variations in 36,213 *G. max* genes and 20,645 in 12,332 *G. soja* genes, resulting in nonsynonymous, stop gain or stop loss mutations. This study provided the first comprehensive understanding of rare‐allele variants in wild and cultivated soybean genomes and its potential impact on gene functions. This information will be valuable for future studies aimed at improving soybean varieties, as these variants may help reveal the underlying mechanisms controlling traits and have the potential to improve stress resistance, yield, and adaptability to environments.

## Core Ideas

- Genetic variants including rare‐allele variants were identified and annotated based on sequence of 1556 accessions.
- Rare‐allele variants clustered in noncoding regions and euchromatic regions and resulted in nonsynonymous or stop gain/stop loss mutations in 40%–60% of genes.
- Domestication greatly reduced the number of variants and allele frequencies in cultivated compared to wild soybean.
- Resources are provided for future biological insights and research to understand how rare‐allele loci affect complex traits.

**Abbreviations**
MAF: minor allele frequency
SNP: single nucleotide polymorphism

## 1 INTRODUCTION

Genetic variants can be classified into three types based on their minor allele frequency (MAF): common genetic variants with MAF > 5%, low‐frequency variants with MAF between 1% and 5%, and rare‐allele variants with MAF < 1% (Nicolae, 2016). Rare‐allele phenomenon was common in plant, animal, and human genomes (Schilthuizen et al., 2001). The 1000 Genomes Project (The 1000 Genomes Project Consortium, 2012) reported the biological characteristics of low‐frequency variants in human populations: On average, 26.87% of gene bins, 35.47% of intergenic bins, 42.85% of pathway bins, 14.86% of ORegAnno regulatory bins, and 5.97% of evolutionarily conserved regions showed statistically significant differences in low‐frequency variant burden across populations. Conserved or functionally relevant regions had fewer significant differences in low‐frequency burden than regions under less evolutionary constraint (Moore et al., 2013). Factors such as rapid population growth and weak purifying selection had lowed ancestral populations to accumulate excessive low‐frequency variants in the genome (Moore et al., 2013).

Rare‐allele variants were likely essential for understanding the etiology of common complex traits and uncovering the mechanisms underlying these traits. Genome‐wide association studies in humans showed that adult height was associated with approximately 700 common variants that together explained approximately 20% of the height heritability (Wood et al., 2014); however, the average effect of 32 rare‐allele variants and 51 low‐frequency (1% < MAF ≤ 5%) variants was 10 times greater than that of common variants (Marouli et al., 2017). Rare‐allele variants might also play a role in complex human disease risk (Keinan & Clark, 2012), for example, low‐frequency missense and rare‐allele nonsense single nucleotide polymorphisms (SNPs) in the lipoprotein gene (Chasman et al., 2009; Mehta, 2011) and proprotein convertase subtilisin/kexin type 9 serine protease gene (Cohen et al., 2006, 2005) had large effects on low density lipoprotein cholesterol and coronary artery disease risk, respectively. Protein‐truncating variants in GPR75 were found in ∼4/10,000 sequenced people and were associated with a 1.8 kg/m^2^ reduction in body mass index, a 5.3 kg weight loss, and a 54% reduction in the odds of obesity in heterozygous carriers (Akbari et al., 2021). Rare‐allele variants often caused monogenic disorders in humans (Hirschhorn & Daly, 2005). Analysis of the whole‐genome sequences from avian IAV H7N9 patients and healthy controls identified a strong association between H7N9 infection and a rare heterozygous single‐nucleotide variant in the *MX1* gene (Chen et al., 2021).

In plants, although the genome‐wide distribution of rare‐allele variants has not yet been reported, there had been some reports on the functions of rare‐allele variants. The *OsTCP19* allele associated with high tillering response to nitrogen was reported to be common in wild rice but was largely lost in modern cultivars (Liu et al., 2021). The *YrAS2388R* allele associated with wheat stripe rust resistance was present in *Aegilops tauschii* Coss., the donor of the common wheat D genome and *A*. *tauschii*–derived synthetic wheat but was absent in 461 common wheat lines tested (C. Zhang et al., 2019). Rare alleles were also shown to be associated with dysregulated expression and correlated with seed‐weight fitness (Kremling et al., 2018). Looking back at the history of plant breeding, the process of plant improvement had also led to some low‐frequency variants with advantages becoming common variants in improved varieties.

Soybean (*Glycine max*) is an important source of protein meal and vegetable oil used for human consumption and animal feed as well as industrial uses such as biofuel (Hartman et al., 2011). Moreover, soybean plays an important role in the production of other crops as it adds nitrogen to the soil during crop rotation (Singh, 2010). In the past decade, many soybean accessions were sequenced (Schmutz et al., 2010), the availability of sequence provided an opportunity to explore rare‐allele variants in soybean on a genome‐wide scale. Q. Song et al. (2015) genotyped 18,480 domesticated soybeans and 1168 wild soybean (*Glycine soja*) accessions with the SoySNP50K BeadChip assay containing more than 50,000 SNPs. The dataset was used to select a diverse core set of soybean accessions for breeding applications, of which approximately 400 cultivated soybean and 50 wild soybean accessions were re‐sequenced (Valliyodan et al., 2021), and resequencing of additional soybean genomes was also reported by others (Bayer et al., 2022; Chung et al., 2014; Fang et al., 2017; Han et al., 2016; Kajiya‐Kanegae et al., 2021; M. Y. Kim et al., 2010; Li et al., 2014; Liu et al., 2020; Maldonado dos Santos et al., 2016; Qi et al., 2021, 2014; Qiu et al., 2014; Shen et al., 2019, 2018; Shimomura et al., 2015; Torkamaneh et al., 2018; Valliyodan et al., 2019, 2016; Xie et al., 2019; Zhou et al., 2015). H. Zhang et al. (2022) consolidated and analyzed the 1556 soybean genomes of wild, landraces, and elite soybean cultivars available in the public domains and generated at USDA‐ARS, Beltsville, MD. Based on this study, we further functionally annotated all variants in the soybean genome (including the rare‐allele variants) according to the gene models, investigated landscape of rare‐allele variants in soybean genome, and compared the rare‐allele frequencies between *G. max* and *G. soja* populations.

**Core Ideas**

- Genetic variants including rare‐allele variants were identified and annotated based on sequence of 1556 accessions.
- Rare‐allele variants clustered in noncoding regions and euchromatic regions and resulted in nonsynonymous or stop gain/stop loss mutations in 40%–60% of genes.
- Domestication greatly reduced the number of variants and allele frequencies in cultivated compared to wild soybean.
- Resources are provided for future biological insights and research to understand how rare‐allele loci affect complex traits.

## 2 MATERIALS AND METHODS

### 2.1 Genotypic data

A total of 1556 soybean whole genome sequences generated at Soybean Genomic Improvement Laboratory, USDA‐ARS, Beltsville, MD, and deposited at SRA NCBI were analyzed and approximately 30 million SNPs were identified (H. Zhang et al., 2022). The accession types, maturity groups, countries of origin, longitude and latitude of the 1556 accessions were previously reported by H. Zhang et al. (2022). These accessions came from 38 countries and their maturity groups ranged from 0 to X. We converted the SNP VCF files to SNP allele files using an in‐house script, and the script also filtered all the SNPs with only one allele and with only heterozygotes. We further eliminated the SNPs with tri‐alleles and with missing and heterozygotes greater than 30%. To reduce false SNP calling, SNPs with the number of accessions containing minor alleles less than or equal to 2 across 1556 cultivated and wild soybeans were eliminated. SNPs with MAF lower than 1% were defined as rare‐allele variants (Auer & Guillaume, 2015; Y. J. Kim et al., 2022; Sazonovs & Barrett, 2018; Q. Wang et al., 2021). Although different thresholds such as 1%, 0.5%, or 0.1% were used in different publications, we adopted the commonly used threshold of 1%. Although number of rare‐allele variants may vary depending on the sample size, a large sample size and a diverse set of germplasm used in this report may minimize the impact.

### 2.2 Annotation of SNPs in the soybean genome

The sequence annotation was performed using Annovar. Reference genome sequence and annotation files from assembly Wm82a2v1 (Gmax_275_v2.0) were used. The reference genome sequence was downloaded from https://data.jgi.doe.gov/refine‐download/phytozome?q=glycine&expanded=Phytozome‐275 (Schmutz et al., 2010) and was converted to Gm_refGeneMrna.fa. The input variant file included the following columns: variant chromosome IDs, variant start and end physical positions, reference genome variant alleles, and alternate variant alleles based on the sequence alignment to Wm82a2v1 assembly.

### 2.3 Selection of insect‐resistant genes

To screen for insect‐resistant genes, we included only those genes that encoded proteins with relatively clear mechanisms of action on insects. Thus, we selected protease inhibitors that inhibited insect digestive proteases (Nunes et al., 2020); plant lectins that were toxic to various insects such as Coleoptera, Diptera, Lepidoptera, Hymenoptera, Neuroptera, and Thysanoptera (Lagarda‐Diaz et al., 2017); amylase inhibitors that inhibited insect amylase (B. Wang et al., 2023); chitinases that were involved in plant defense (Vaghela et al., 2022); and NBS‐LRR proteins that played a key role in plant defense against biotic stresses (Pirithiraj et al., 2023).

## 3 RESULTS

### 3.1 The identification and annotation of variants

A total of 13,651,475 and 16,505,265 variants were identified in *G. max* and *G. soja* populations, respectively. Of the 13,651,475 variants in the *G. max*, 6,533,419 were rare‐allele variants. Of the 16,505,265 variants in the *G. soja* population, 941,274 were rare‐allele variants. All the rare‐allele variants were positioned in different genic regions: downstream, exonic, exonic‐splicing, intergenic, intronic, upstream, 3′ UTR, and 5′ UTR, and the type of mutations was also annotated and listed in Tables S1 and S2. The dataset containing annotations of all variants with rare alleles, low‐frequency alleles, and common alleles has been deposited at the Soybase site (https://data.soybase.org/Glycine/max/diversity/Wm82.gnm2.div.Liu_Shi_2025/) for public access.

### 3.2 The distribution of rare‐allele variants on chromosomes

**FIGURE 1:**
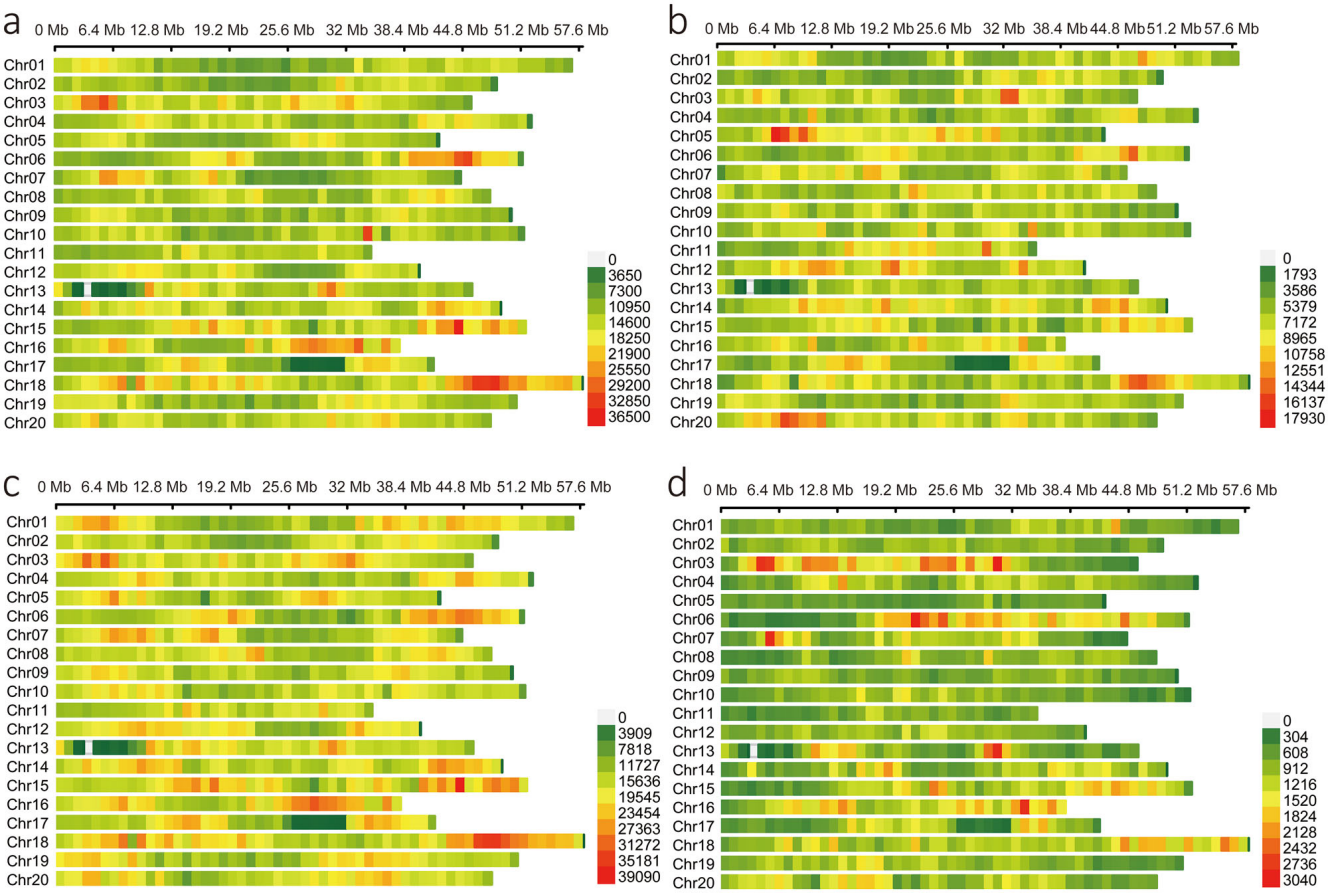
Distribution of variants on chromosomes. (a) Distribution of all variants identified on the 20 chromosomes of *G. max*. (b) Distribution of rare‐allele variants identified on the 20 chromosomes of *G. max*. (c) Distribution of all variants identified on the 20 chromosomes of *G. soja*. (d) Distribution of rare‐allele variants identified on the 20 chromosomes of *G. soja*.

In the *G. max* population, the average number of rare‐allele variants per chromosome was 326,671 and ranged from 250,081 (Chr11) to 427,813 (Chr18). Rare‐allele variant density ranged from 5467 (Chr13) to 8466 (Chr05) per 1 Mb, with an average of 6913. In the *G. soja* population, the average number of rare‐allele variants per chromosome was 47,064, ranging from 24,496 (Chr11) to 72,171 (Chr18). The density of rare‐allele variants per 1 Mb window ranged from 636 (Chr05) to 1477 (Chr03), with an average of 989 (Figure 1; Table S3). Although the total number of variants was 20% higher in *G. soja* than *G. max* (16,505,265 in *G. soja* vs. 13,651,475 in *G. max*), the number of rare‐allele variants in *G. soja* was only 14% of the number in *G. max*. Rare‐allele variants accounted for 5.7% of all SNPs in the *G. soja* population and 47.9% in the *G. max* population. These findings highlighted the abundance of rare‐allele variants in soybean and the significant differences in the prevalence of rare‐allele variants between *G. max* and *G. soja* populations. The rare‐allele variants in the heterochromatic region of the *G. max* and *G. soja* population accounted for 53.33% and 58.61% of all rare‐allele variants, respectively, which were slightly higher than the proportion of the heterochromatic region in the genome (52.82%) (Table 1).

**TABLE 1:** Distribution of rare‐allele variants in heterochromatic and euchromatic regions.

Chromosome	Heterochromatic region (Mb)	Number and percentage of rare‐allele variants in heterochromatic region of the *G. max* population	Number and percentage of rare‐allele variants in euchromatic region of the *G. max* population	Number and percentage of rare‐allele variants in heterochromatic region of the *G. soja* population	Number and percentage of rare‐allele variants in euchromatic region of the *G. soja* population
Chr01	8.1–47.4	234,075 (64.09%)	131,162 (35.91%)	33,685 (72.62%)	12,700 (27.38%)
Chr02	16.0–38.2	133,858 (47.29%)	149,199 (52.71%)	20,622 (47.89%)	22,442 (52.11%)
Chr03	6.9–33.4	190,719 (58.82%)	133,536 (41.18%)	48,294 (71.42%)	19,329 (28.58%)
Chr04	10.4–43.5	198,573 (62.00%)	121,728 (38.00%)	36,469 (71.79%)	14,328 (28.21%)
Chr05	6.4–30.2	238,395 (66.68%)	119,104 (33.32%)	14,930 (55.59%)	11,926 (44.41%)
Chr06	18.2–44.4	189,325 (53.51%)	164,477 (46.49%)	47,345 (70.55%)	19,759 (29.45%)
Chr07	17.7–34.6	105,130 (33.69%)	206,929 (66.31%)	20,345 (44.37%)	25,505 (55.63%)
Chr08	22.9–40.4	127,990 (39.56%)	195,544 (60.44%)	15,700 (40.10%)	23,454 (59.90%)
Chr09	6.4–38.8	210,790 (68.18%)	98,360 (31.82%)	26,779 (67.80%)	12,716 (32.20%)
Chr10	6.9–36.9	203,952 (60.93%)	130,771 (39.07%)	28,842 (68.91%)	13,014 (31.09%)
Chr11	11.4–30.0	162,507 (64.98%)	87,574 (35.02%)	15,520 (63.36%)	8,976 (36.64%)
Chr12	8.2–32.4	207,491 (62.88%)	122,473 (37.12%)	24,860 (66.96%)	12,265 (33.04%)
Chr13	0–13.3	45,240 (18.04%)	205,532 (81.96%)	10,928 (26.29%)	30,638 (73.71%)
Chr14	9.7–43.7	294,059 (72.93%)	109,168 (27.07%)	37,715 (75.40%)	12,307 (24.60%)
Chr15	18.3–43.0	144,765 (40.58%)	212,004 (59.42%)	32,373 (54.78%)	26,726 (45.22%)
Chr16	8.3–26.8	124,406 (44.96%)	152,292 (55.04%)	25,669 (47.82%)	28,013 (52.18%)
Chr17	14.3–35.8	123,672 (47.82%)	134,925 (52.18%)	22,761 (61.56%)	14,213 (38.44%)
Chr18	20.5–43.3	134,809 (31.51%)	293,004 (68.49%)	25,665 (35.56%)	46,506 (64.44%)
Chr19	8.9–34.3	141,051 (44.80%)	173,768 (55.20%)	27,547 (59.00%)	19,145 (41.00%)
Chr20	3.2–33.7	273,543 (71.78%)	107,519 (28.22%)	35,629 (69.50%)	15,634 (30.50%)
Total	501.4 (52.82%)	3,484,350 (53.33%)	3,049,069 (46.67%)	551,678 (58.61%)	389,596 (41.39%)

### 3.3 Distribution of rare‐allele variants identified from *G. max* and *G. soja* populations across gene structures

**FIGURE 2:**
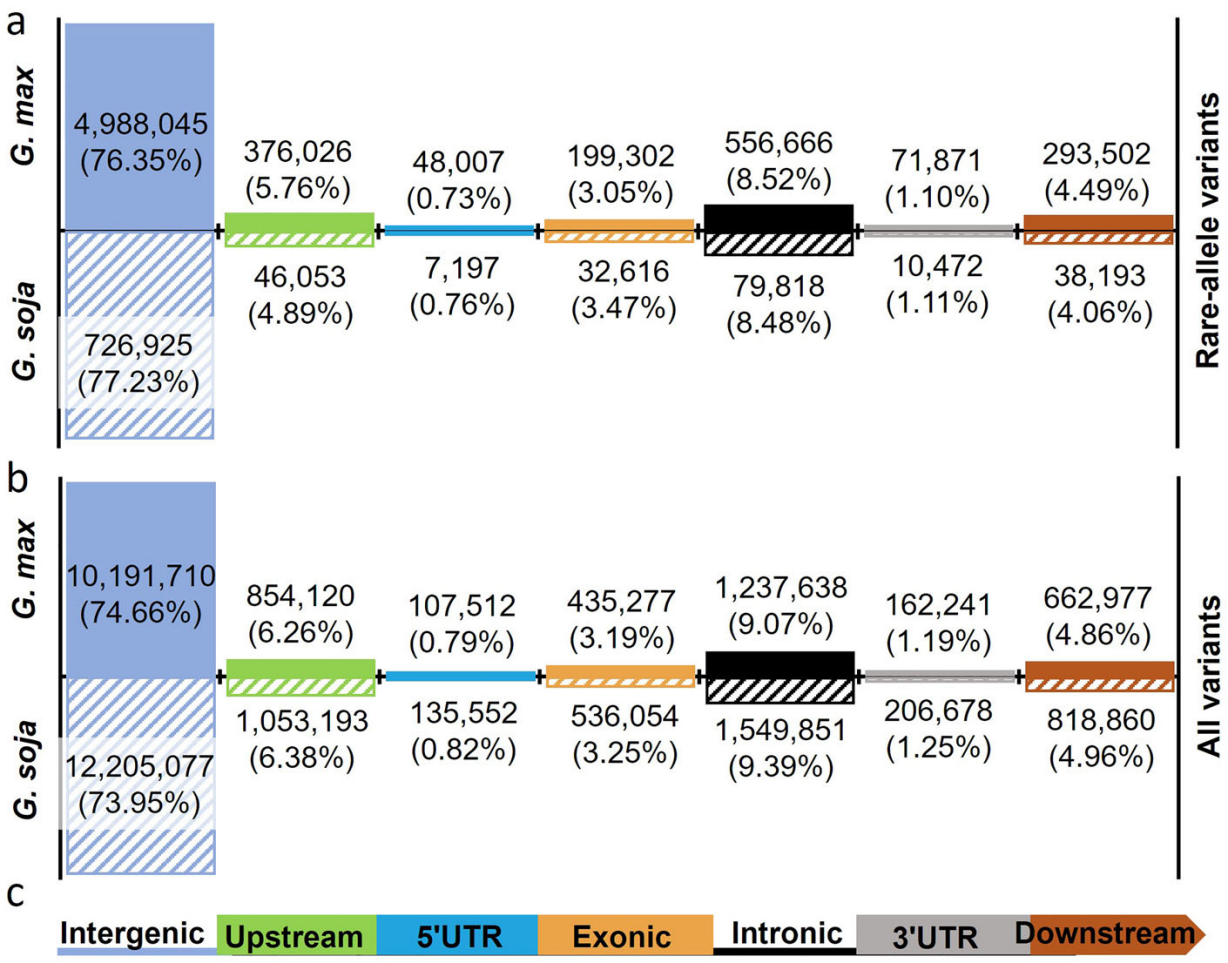
Distribution of variants identified from the *G. max* and *G. soja* populations across various gene structures. (a) Distribution of rare‐allele variants identified in *G. max* and *G. soja* in different gene structures. (b) Distribution of all variants identified in *G. max* and *G. soja* in different gene structures. The height of solid boxes and diagonal lines in (a) and (b) represent proportions of variants identified in the *G. max* and *G. soja* populations, respectively. (c) Gene structures. Blue line represents intergenic region, green box upstream region, blue box 5′ UTR, yellow box exonic region, black line intronic region, gray box 3′ UTR, and brown narrow downstream region.

In *G. max* population, the 13,651,475 variants were placed in different genomic regions (Figure 2; Table S4). The numbers and proportions of variants in different regions were as follows: downstream 662,977 (4.8%), exons 431,588 (3.1%), intergenic regions 10,191,710 (74.6%), introns 1,237,638 (9.0%), upstream 794,429 (5.8%), 3′ UTR 162,241 (1.1%), and 5′ UTR 107,293 (0.7%). In the *G. soja* population, the 16,505,265 variants were also annotated (Figure 2; Table S4): downstream 818,860 (4.9%), exons 531,478 (3.2%), intergenic regions 12,205,077 (73.9%), introns 1,549,851 (9.3%), upstream 978,973 (5.9%), 3′ UTR 206,678 (1.2%), and 5′ UTR 135,247 (0.8%).

For rare‐allele variants, a total of 4,988,045 (76.35%) and 1,545,374 (23.65%) variants from the *G. max* population were in intergenic regions and genes, respectively. Of all the intragenic variants, 376,026 (24.33%), 48,007 (3.10%), 199,302 (12.89%), 556,666 (36.02%), 71,871 (4.65%), and 293,502 (18.99%) were in upstream, 5′ UTR, exon, intron, 3′ UTR, and downstream regions of genes, respectively. In *G. soja* population, a total of 726,925 (77.23%) and 214,349 (22.77%) rare‐allele variants were in intergenic regions and genes, respectively. Among the variants within genes, 46,053 (21.49%), 7,197 (3.36%), 32,616 (15.21%), 79,818 (37.24%), 10,472 (4.89%), and 38,193 (17.82%) were in upstream, 5′ UTR, exon, intron, 3′ UTR, and downstream regions of genes, respectively (Figure 2).

### 3.4 The proportion of variants causing amino acid changes among rare‐allele and non‐rare allele variants

In the *G. max* population, a total of 7,118,056 non‐rare allele variants were identified, of which 136,526 (1.92%) caused missense mutations, stop gain mutations, or stop loss mutations, and 121,450 (1.86%) of the 6,533,419 rare‐allele variants caused such mutations in 36,213 genes. In the *G. soja* population, a total of 16,505,265 variants were identified, of which 292,301 (1.88%) caused missense mutations and stop gain/stop loss mutations. A total of 20,645 (2.19%) of the 941,274 rare‐allele variants caused such mutations in 12,332 genes (Tables S1, S2, and S4).

### 3.5 The MAF comparison of variants between *G. soja* and *G. max* populations

**FIGURE 3:**
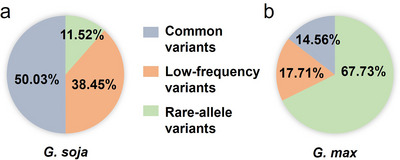
Comparison of minor allele frequencies (MAF) of rare‐allele variants between *G. soja* and *G. max* populations. (a) MAF of 5,281,690 *G. max* rare‐allele variants in *G. soja* population. (b) MAF of 898,229 *G. soja* rare‐allele variants in *G. max* population. Light blue represents common variants, orange represents low‐frequency variants, and light green represents rare‐allele variants.

Among the 6,533,419 rare‐allele variants identified in *G. max*, 5,281,690 were shared between the *G. max* and *G. soja* populations. MAF analysis of the shared variants in the *G. soja* population showed that 608,392 variants were also with rare alleles, accounting for 11.25%; 2,030,674 variants were low‐frequency, accounting for 38.45%; and 2,642,624 variants were common, accounting for 50.03% in the *G. soja* (Figure 3). These results indicated that most of the rare‐allele variants in the *G. max* population were due to the decreased MAF of low‐frequency and common variants in the *G. soja* population (71.53%), and 19.16% of the rare‐allele variants were due to new mutations arising in the *G. max* population.

For the 941,274 rare‐allele variants identified in the *G. soja* population, 898,229 variants were shared in both the *G. max* and *G. soja* populations. Analysis of these shared variants in the *G. max* population showed that 608,392 (67.7%) variants were rare, 159,077 (17.7%) were low‐frequency, and 130,760 (14.5%) were common in the *G. max* population (Figure 3). Most (64.63%) rare‐allele variants from the *G. soja* population remained in the *G. max* population, and the remaining rare‐allele variants (30.97%) were converted into low‐frequency or common variants.

### 3.6 The ratio of transitions and transversions of the variants

**FIGURE 4:**
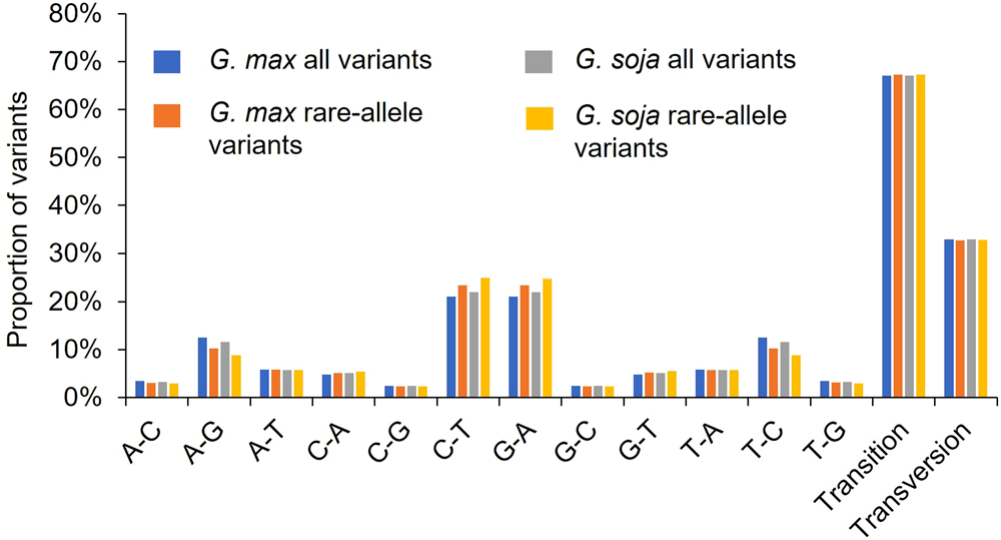
Ratio of transitions and transversions of the variants. Horizontal axis represents the types of nucleotide substitutions, and vertical axis represents the proportion of various nucleotide substitutions. The blue bars represent all variants in *G. max* population, orange bars represent rare‐allele variations in *G. max* population, gray bars represent all variants in *G. soja* population, and yellow bars represent rare‐allele variants in *G. soja* population.

Base substitution analysis of all variants and rare‐allele variants in *G. max* and *G. soja* populations revealed the presence of 12 mutations. The proportions of different forms of base substitutions did not differ much between *G. max* and *G. soja* populations and between all variants and rare‐allele variants. Among the 12 base substitutions, C‐T and G‐A were the two most common substitution types, accounting for 21.03% and 24.85% of all substitutions, respectively, whereas C‐G and G‐C were the least common substitution types, accounting for 2.28% and 2.40% of all substitutions, respectively. These base substitutions can be divided into transitions and transversions, with transitions accounting for 67% of all substitutions and transversions accounting for 33% in both populations, all variants and rare‐allele variants categories (Figure 4).

### 3.7 Distribution of rare‐allele variants in insect‐resistant genes—An example

**FIGURE 5:**
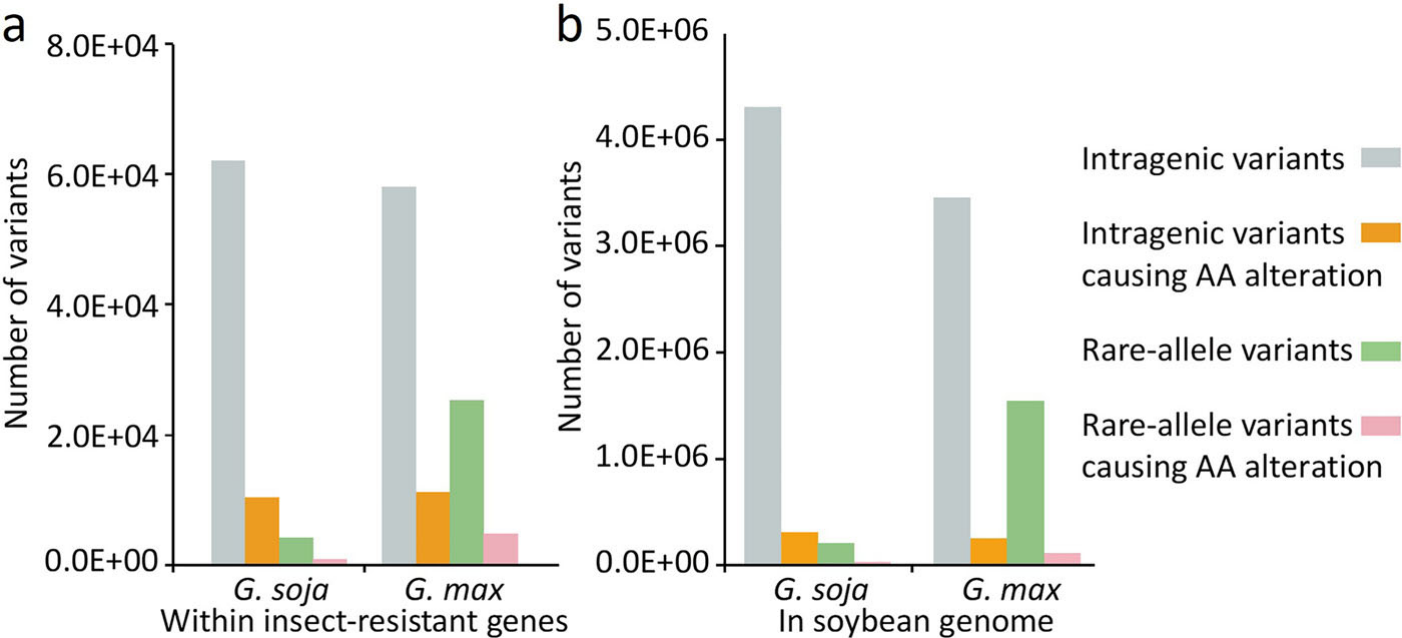
Number of variants in insect‐resistant genes and whole genome. (a) Number of variants in insect‐resistant genes identified in *G. max* and *G. soja*. (b) Number of variants in whole genome of *G. max* and *G. soja*. The gray bars represent intragenic variants in the *G. max* and *G. soja* populations, orange bars represent intragenic variants causing amino acid alteration, green bars represent intragenic rare‐allele variants, and pink bars represent intragenic rare‐allele variants causing amino acid alteration.

In the soybean genome annotation file, we searched for genes annotated as proteinase inhibition, phytolection, amylase inhibition, chitinase, and NBS‐LRR, and obtained 45, 255, 71, 44, and 258 genes, respectively, for a total of 673 genes (Table S5). There were 62,260 variants within the intragenic regions of these insect‐resistant genes of *G. soja*, of which 10,418 variants resulted in amino acid changes. Among the 62,260 variants, 4065 were rare‐allele variants and were distributed in 580 insect‐resistant genes, 863 of the 4065 rare‐allele variants led to amino acid changes in 273 of these genes. There were 58,217 variants located in the intragenic regions of these genes of *G. max*, of which 11,070 variants led to amino acid changes. Among the 58,217 variants, 25,229 were rare‐allele variants, of which 4841 caused amino acid changes. There were 25,229 rare‐allele variants in 600 insect‐resistant genes, of which 539 genes contained at least one rare‐allele variant that caused amino acid changes. We compared the numbers of all variants, rare‐allele variants, and variants causing amino acid changes in the intragenic regions in both *G. soja* and *G. max* populations, across the genome and in the insect‐resistant genes. The results indicated that across the entire genome, the number of intragenic variants causing amino acid changes was 7/100, and the number of intragenic rare‐allele variants causing amino acid changes was 1/10 in *G. soja* and 8/100 in *G. max*. However, within insect‐resistant genes, these values were approximately 2.4 times those observed genome‐wide (Figure 5). The enrichment of amino‐alternation mutations in rare‐allele variants in biotic stress resistance genes may be a result of soybean's response to frequent changes in insect species and insect population sizes during its long domestication history.

## 4 DISCUSSION

### 4.1 Multiple factors influence the distribution of rare‐allele variants on chromosomes

By observing the chromosomal distribution of rare‐allele variants and all variants (including common, low‐frequency, and rare‐allele variants) identified in *G. max* and *G. soja* populations (Figure 1), we found that in *G. max* and *G. soja* populations, the number of all variants and rare‐allele variants located in heterochromatin regions was higher than that of variants located in euchromatin regions (Table 1; Figure 1), and the number of rare‐allele variants in noncoding regions was higher than that of variants in coding regions. The higher proportion of rare‐allele variants in heterochromatic regions might be caused by a combination of factors such as reduced selective pressure, high mutation rate, low recombinant rate, and genetic drift. Heterochromatic regions often contained repetitive sequences, satellite DNA, and fewer protein‐coding genes. These regions typically experienced less selective pressure compared to euchromatic regions, where functional genes were more prevalent. This reduced selective pressure allowed for a higher accumulation of genetic variants, including those with rare alleles. Studies have shown that some heterochromatin regions had higher mutation rates than euchromatin regions. This higher mutation rate might lead to an increase in the number of genetic variations and rare alleles in these regions. Heterochromatic regions also had lower rates of recombination that played a key role in mixing genetic material and could reduce the frequency of minor alleles in euchromatic regions through mechanisms such as genetic drift and selection. In heterochromatic regions with lower recombination, minor alleles might persist at higher frequencies over evolutionary timescales. Noncoding regions generally had a higher proportion of variants with rare alleles compared to coding regions for several reasons: Exons coded for proteins, and any variations in these regions could have a direct impact on protein function. Therefore, deleterious mutations in coding regions were often removed by purifying selection, leading to a lower frequency of variants. In contrast, variants in noncoding regions were more likely to be neutral; neutral variants were not subject to strong selective pressures and could drift in the population, leading to a higher proportion of rare alleles.

In *G. max* population, dense clusters of rare‐allele variants observed on Chromosomes 3, 5, 6, 18, and 20, some of these densely clustered hotspots (e.g., regions on Chromosomes 5 and 20) did not have a large number of total variants. Although the reasons for the formation of most, if not all, rare‐allele variant clusters were unclear, the cluster formation of a few rare‐allele variants could be inferred from the annotated gene functions in *Arabidopsis thaliana*, for example, the rare‐allele variant G/A at Chr05_7237778 on Chromosome 5, whose genotype “AA” was 0.60% in cultivated soybean but 55.50% in wild soybean, dropped sharply during domestication and improvement (Figure S1). This might be attributed to the functionality of the gene (*SMO1*) containing the variant. In *Arabidopsis*, double mutations within this gene could cause embryonic lethality (J. Song et al., 2019). In cultivated soybean, some new double mutations within this gene might also cause embryonic lethality and led to low survival of specific genotypes, and the clustering of rare‐allele variants might be expanded due to the extent of linkage disequilibrium in this genomic region containing the *SMO1* gene.

### 4.2 Domestication and selection led to a reduction in genetic diversity, resulting in the loss of variants and increased rare‐allele variants in cultivated soybean

Our results showed that domestication and artificial selection had reduced the MAF of most non‐rare variants from *G. soja* population. The number of variants in cultivated soybean was 20% lower than in wild soybean, and the number of rare‐allele variants in cultivated soybean was six times higher than in wild soybean.

This suggested that domestication and selection had greatly reduced the genetic diversity of the cultivated soybean by reducing the total number of variants and decreasing variant allele frequency. The reduction of genetic diversity might have led to an increased vulnerability of soybean to biotic and abiotic stresses. These findings were consistent with the view that a genetic diversity bottleneck occurred during soybean domestication and improvement, and that wild soybean retained allelic diversity appeared to have been lost in cultivated soybean (Hyten et al., 2006; Lam et al., 2010). Domestication and selection can also increase the proportion of rare‐allele variants in cultivated soybean compared to wild soybean due to several evolutionary and genetic mechanisms. In the wild, natural selection removes deleterious alleles that reduce survival or reproduction. Under domestication, human care (e.g., irrigation, fertilizers, and pest control) reduces natural selective pressures. As a result, some rare deleterious mutations that would have been eliminated in the wild can persist or even increase in frequency. Occasionally, cultivated soybeans may hybridize with wild relatives or other cultivated lines, introducing new rare alleles. If these alleles are not immediately selected against, they may persist. Neutral mutations may also play an important role for the presence of rare‐allele variants in cultivated soybean.

### 4.3 Significance of variant functional annotation

Genome‐wide functional annotation of genetic variation, especially rare‐allele variants, can play a key role in unraveling the mysteries of plant genomes. These rare‐allele variants were often overlooked due to their low frequency, but they can carry key adaptive traits such as enhanced stress tolerance or improved yield. Information of the identified variants that alter protein amino acids, function, or even completely stop protein production throughout the soybean genome will help to determine their functions, understand how they affect complex traits, and aid in soybean improvement.

## AUTHOR CONTRIBUTIONS

**Zhi Liu**: Formal analysis; investigation; writing—original draft. **Xiaolei Shi**: Investigation. **Qing Yang**: Investigation. **Ying Li**: Investigation. **Chunyan Yang**: Investigation. **Mengchen Zhang**: Investigation. **Yong‐Qiang Charles An**: Resources; writing—review and editing. **Henry T. Nguyen**: Resources; writing—review and editing. **Long Yan**: Conceptualization; formal analysis; funding acquisition; investigation; writing—original draft. **Qijian Song**: Conceptualization; formal analysis; investigation; methodology; project administration; resources; writing—original draft; writing—review and editing.

## CONFLICT OF INTEREST STATEMENT

The authors declare no conflicts of interest.

## Supporting information



Table S1. Rare‐allele variants in *G. max* that cause nonsynonymous, stop‐gain and stop‐loss mutations.

Table S2. Rare‐allele variants in *G. soja* that cause nonsynonymous, start‐loss, stop‐gain and stop‐loss mutations.

Table S3. Distribution of all variants and rare‐allele variants on chromosomes.

Table S4. Proportion of variants causing amino acid changes among rare‐allele and non‐rare allele variants.

Table S5. A list of insect‐resistant genes based on the Wm82a2v1 assembly annotation.

Figure S1. Allele frequencies of the variants at Chr05_7237778 in *G. soja* and *G. max* populations. Blue represents the soybean accessions with the “A” allele, orange represents the accessions with the “G” allele.

## Data Availability

The genotypic dataset for the diverse set of wild and cultivated soybean is available at Ag Data Commons (https://doi.org/10.15482/USDA.ADC/1519167). The dataset containing annotations for all the variants with rare alleles, low‐frequency alleles, or common alleles has been deposited at the public available site Soybase (https://data.soybase.org/Glycine/max/diversity/Wm82.gnm2.div.Liu_Shi_2025/). The annotations for rare‐allele variants in *G. max* and *G. soja* are in Tables S1 and S2.

## References

[tpg270020-bib-0001] Akbari, P. , Gilani, A. , Sosina, O. , Kosmicki, J. A. , Khrimian, L. , Fang, Y. Y. , Persaud, T. , Garcia, V. , Sun, D. , Li, A. , Mbatchou, J. , Locke, A. E. , Benner, C. , Verweij, N. , Lin, N. , Hossain, S. , Agostinucci, K. , Pascale, J. V. , Dirice, E. , … Lotta, L. A. (2021). Sequencing of 640,000 exomes identifies *GPR75* variants associated with protection from obesity. Science, 373, eabf8683. 10.1126/science.abf8683 34210852 PMC10275396

[tpg270020-bib-0002] Auer, P. , & Guillaume, L. (2015). Rare variant association studies: Considerations, challenges and opportunities. Genome Medicine, 7, 1–11. 10.1186/s13073-015-0138-2 25709717 PMC4337325

[tpg270020-bib-0003] Bayer, P. E. , Valliyodan, B. , Hu, H. , Marsh, J. I. , Yuan, Y. , Vuong, T. D. , Patil, G. , Song, Q. , Batley, J. , Varshney, R. K. , Lam, H. M. , Edwards, D. , & Nguyen, H. T. (2022). Sequencing the USDA core soybean collection reveals gene loss during domestication and breeding. The Plant Genome, 15(1), e20109. 10.1002/tpg2.20109 34169673 PMC12807262

[tpg270020-bib-0004] Chasman, D. I. , Shiffman, D. , Zee, R. Y. , Louie, J. Z. , Luke, M. M. , Rowland, C. M. , Catanese, J. J. , Buring, J. E. , Devlin, J. J. , & Ridker, P. M. (2009). Polymorphism in the apolipoprotein(a) gene, plasma lipoprotein(a), cardiovascular disease, and low‐dose aspirin therapy. Atherosclerosis, 203, 371–376. 10.1016/j.atherosclerosis.2008.07.019 18775538 PMC2678922

[tpg270020-bib-0005] Chen, Y. , Graf, L. , Chen, T. , Liao, Q. , Bai, T. , Petric, P. P. , Zhu, W. , Yang, L. , Dong, J. , Lu, J. , Chen, Y. , Shen, J. , Haller, O. , Staeheli, P. , Kochs, G. , Wang, D. , Schwemmle, M. , & Shu, Y. (2021). Rare variant *MX1* alleles increase human susceptibility to zoonotic H7N9 influenza virus. Science, 373, 918–922. 10.1126/science.abg5953 34413236

[tpg270020-bib-0006] Chung, W. H. , Jeong, N. , Kim, J. , Lee, W. K. , Lee, Y. G. , Lee, S. H. , Yoon, W. , Kim, J. H. , Choi, I. Y. , Choi, H. K. , Moon, J. K. , Kim, N. , & Jeong, S. C. (2014). Population structure and domestication revealed by high‐depth resequencing of Korean cultivated and wild soybean genomes. DNA Research, 21, 153–167. 10.1093/dnares/dst047 24271940 PMC3989487

[tpg270020-bib-0007] Cohen, J. C. , Boerwinkle, E. , Mosley Jr, T. H. , & Hobbs, H. H. (2006). Sequence variations in PCSK9, low LDL, and protection against coronary heart disease. The New England Journal of Medicine, 354, 1264–1272. 10.1056/NEJMoa054013 16554528

[tpg270020-bib-0008] Cohen, J. , Pertsemlidis, A. , Kotowski, I. K. , Graham, R. , Garcia, C. K. , & Hobbs, H. H. (2005). Low LDL cholesterol in individuals of African descent resulting from frequent nonsense mutations in PCSK9. Nature Genetics, 37, 161–165. 10.1038/ng1509 15654334

[tpg270020-bib-0009] Fang, C. , Ma, Y. , Wu, S. , Liu, Z. , Wang, Z. , Yang, R. , Hu, G. , Zhou, Z. , Yu, H. , Zhang, M. , Pan, Y. , Zhou, G. , Ren, H. , Du, W. , Yan, H. , Wang, Y. , Han, D. , Shen, Y. , Liu, S. , … Tian, Z. (2017). Genome‐wide association studies dissect the genetic networks underlying agronomical traits in soybean. Genome Biology, 18, 161. 10.1186/s13059-017-1289-9 28838319 PMC5571659

[tpg270020-bib-0010] Han, Y. , Zhao, X. , Liu, D. , Li, Y. , Lightfoot, D. A. , Yang, Z. , Zhao, L. , Zhou, G. , Wang, Z. , Huang, L. , Zhang, Z. , Qiu, L. , Zheng, H. , & Li, W. (2016). Domestication footprints anchor genomic regions of agronomic importance in soybeans. The New Phytologist, 209, 871–884. 10.1111/nph.13626 26479264

[tpg270020-bib-0011] Hartman, G. L. , West, E. D. , & Herman, T. K. (2011). Crops that feed the World 2. Soybean—Worldwide production, use, and constraints caused by pathogens and pests. Food Security, 3, 5–17. 10.1007/s12571-010-0108-x

[tpg270020-bib-0012] Hirschhorn, J. N. , & Daly, M. J. (2005). Genome‐wide association studies for common diseases and complex traits. Nature Reviews Genetics, 6, 95–108. 10.1038/nrg1521 15716906

[tpg270020-bib-0013] Hyten, D. L. , Song, Q. , Zhu, Y. , Choi, I. Y. , Nelson, R. L. , Costa, J. M. , Specht, J. E. , Shoemaker, R. C. , & Cregan, P. B. (2006). Impacts of genetic bottlenecks on soybean genome diversity. Proceedings of the National Academy of Sciences of the United States of America, 103, 16666–16671. 10.1073/pnas.0604379103 17068128 PMC1624862

[tpg270020-bib-0014] Kajiya‐Kanegae, H. , Nagasaki, H. , Kaga, A. , Hirano, K. , Ogiso‐Tanaka, E. , Matsuoka, M. , Ishimori, M. , Ishimoto, M. , Hashiguchi, M. , Tanaka, H. , Akashi, R. , Isobe, S. , & Iwata, H. (2021). Whole‐genome sequence diversity and association analysis of 198 soybean accessions in mini‐core collections. DNA Research, 28, dsaa032. 10.1093/dnares/dsaa032 33492369 PMC7934572

[tpg270020-bib-0015] Keinan, A. , & Clark, A. G. (2012). Recent explosive human population growth has resulted in an excess of rare genetic variants. Science, 336, 740–743. 10.1126/science.1217283 22582263 PMC3586590

[tpg270020-bib-0016] Kim, M. Y. , Lee, S. , Van, K. , Kim, T. H. , Jeong, S. C. , Choi, I. Y. , Kim, D. S. , Lee, Y. S. , Park, D. , Ma, J. , Kim, W. Y. , Kim, B. C. , Park, S. , Lee, K. A. , Kim, D. H. , Kim, K. H. , Shin, J. H. , Jang, Y. E. , Kim, K. D. , … Lee, S. H. (2010). Whole‐genome sequencing and intensive analysis of the undomesticated soybean (*Glycine soja* Sieb. and Zucc.) genome. Proceedings of the National Academy of Sciences of the United States of America, 107, 22032–22037. 10.1073/pnas.1009526107 21131573 PMC3009785

[tpg270020-bib-0017] Kim, Y. J. , Moon, S. , Hwang, M. Y. , Han, S. , Jang, H. M. , Kong, J. , Shin, D. M. , Yoon, K. , Kim, S. M. , Lee, J. E. , Mahajan, A. , Park, H. Y. , McCarthy, M. I. , Cho, Y. S. , & Kim, B. J. (2022). The contribution of common and rare genetic variants to variation in metabolic traits in 288,137 East Asians. Nature Communications, 13(1), 6642. 10.1038/s41467-022-34163-2 PMC9636136 36333282

[tpg270020-bib-0018] Kremling, K. A. G. , Chen, S. Y. , Su, M. H. , Lepak, N. K. , Romay, M. C. , Swarts, K. L. , Lu, F. , Lorant, A. , Bradbury, P. J. , & Buckler, E. S. (2018). Dysregulation of expression correlates with rare‐allele burden and fitness loss in maize. Nature, 555, 520–523. 10.1038/nature25966 29539638

[tpg270020-bib-0019] Lagarda‐Diaz, I. , Guzman‐Partida, A. M. , & Vazquez‐Moreno, L. (2017). Legume lectins: Proteins with diverse applications. International Journal of Molecular Sciences, 18, 1242. 10.3390/ijms18061242 28604616 PMC5486065

[tpg270020-bib-0020] Lam, H. M. , Xu, X. , Liu, X. , Chen, W. , Yang, G. , Wong, F. L. , Li, M. W. , He, W. , Qin, N. , Wang, B. , Li, J. , Jian, M. , Wang, J. , Shao, G. , Wang, J. , Sun, S. S. , & Zhang, G. (2010). Resequencing of 31 wild and cultivated soybean genomes identifies patterns of genetic diversity and selection. Nature Genetics, 42, 1053–1059. 10.1038/ng.715 21076406

[tpg270020-bib-0021] Li, Y. H. , Zhou, G. , Ma, J. , Jiang, W. , Jin, L. G. , Zhang, Z. , Guo, Y. , Zhang, J. , Sui, Y. , Zheng, L. , Zhang, S. S. , Zuo, Q. , Shi, X. H. , Li, Y. F. , Zhang, W. K. , Hu, Y. , Kong, G. , Hong, H. L. , Tan, B. , … Qiu, L. J. (2014). De novo assembly of soybean wild relatives for pan‐genome analysis of diversity and agronomic traits. Nature Biotechnology, 32, 1045–1052. 10.1038/nbt.2979 25218520

[tpg270020-bib-0022] Liu, Y. , Du, H. , Li, P. , Shen, Y. , Peng, H. , Liu, S. , Zhou, G. A. , Zhang, H. , Liu, Z. , Shi, M. , Huang, X. , Li, Y. , Zhang, M. , Wang, Z. , Zhu, B. , Han, B. , Liang, C. , & Tian, Z. (2020). Pan‐genome of wild and cultivated soybeans. Cell, 182, 162–176.e13. 10.1016/j.cell.2020.05.023 32553274

[tpg270020-bib-0023] Liu, Y. , Wang, H. , Jiang, Z. , Wang, W. , Xu, R. , Wang, Q. , Zhang, Z. , Li, A. , Liang, Y. , Ou, S. , Liu, X. , Cao, S. , Tong, H. , Wang, Y. , Zhou, F. , Liao, H. , Hu, B. , & Chu, C. (2021). Genomic basis of geographical adaptation to soil nitrogen in rice. Nature, 590, 600–605. 10.1038/s41586-020-03091-w 33408412

[tpg270020-bib-0024] Maldonado dos Santos, J. V. , Valliyodan, B. , Joshi, T. , Khan, S. M. , Liu, Y. , Wang, J. , Vuong, T. D. , de Oliveira, M. F. , Marcelino‐Guimarães, F. C. , Xu, D. , Nguyen, H. T. , & Abdelnoor, R. V. (2016). Evaluation of genetic variation among Brazilian soybean cultivars through genome resequencing. BMC Genomics, 17, 110. 10.1186/s12864-016-2431-x 26872939 PMC4752768

[tpg270020-bib-0025] Marouli, E. , Graff, M. , Medina‐Gomez, C. , Lo, K. S. , Wood, A. R. , Kjaer, T. R. , Fine, R. S. , Lu, Y. , Schurmann, C. , Highland, H. M. , Rüeger, S. , Thorleifsson, G. , Justice, A. E. , Lamparter, D. , Stirrups, K. E. , Turcot, V. , Young, K. L. , Winkler, T. W. , Esko, T. , … Lettre, G. (2017). Rare and low‐frequency coding variants alter human adult height. Nature, 542, 186–190. 10.1038/nature21039 28146470 PMC5302847

[tpg270020-bib-0026] Mehta, N. N. (2011). Large‐scale association analysis identifies 13 new susceptibility loci for coronary artery disease. Circulation: Cardiovascular Genetics, 4, 327–329. 10.1161/CIRCGENETICS.111.960443 21673312 PMC3125595

[tpg270020-bib-0027] Moore, C. B. , Wallace, J. R. , Wolfe, D. J. , Frase, A. T. , Pendergrass, S. A. , Weiss, K. M. , & Ritchie, M. D. (2013). Low frequency variants, collapsed based on biological knowledge, uncover complexity of population stratification in 1000 genomes project data. PLoS Genetics, 9, e1003959. 10.1371/journal.pgen.1003959 24385916 PMC3873241

[tpg270020-bib-0028] Nicolae, D. L. (2016). Association tests for rare variants. Annual Review of Genomics and Human Genetics, 17, 117–130. 10.1146/annurev-genom-083115-022609 27147090

[tpg270020-bib-0029] Nunes, N. N. S. , Ferreira, R. S. , de Sá, L. F. R. , de Oliveira, A. E. A. , & Oliva, M. L. V. (2020). A novel cysteine proteinase inhibitor from seeds of *Enterolobium contortisiliquum* and its effect on *Callosobruchus maculatus* larvae. Biochemistry and Biophysics Reports, 25, 100876. 10.1016/j.bbrep.2020.100876 33364447 PMC7750491

[tpg270020-bib-0030] Pirithiraj, U. , Murugan, M. , Jayakanthan, M. , Boopathi, N. M. , Balasubramani, V. , Premalatha, N. , Ramakrishnan, S. H. , & Babu, S. S. (2023). Genome wide identification and evolutionary analysis of vat like NBS‐LRR genes potentially associated with resistance to aphids in cotton. Genetica, 151, 119–131. 10.1007/s10709-023-00181-1 36717534

[tpg270020-bib-0031] Qi, X. , Jiang, B. , Wu, T. , Sun, S. , Wang, C. , Song, W. , Wu, C. , Hou, W. , Song, Q. , Lam, H.‐M. , & Han, T. (2021). Genomic dissection of widely planted soybean cultivars leads to a new breeding strategy of crops in the post‐genomic era. The Crop Journal, 9, 1079–1087. 10.1016/j.cj.2021.01.001

[tpg270020-bib-0032] Qi, X. , Li, M. W. , Xie, M. , Liu, X. , Ni, M. , Shao, G. , Song, C. , Kay‐Yuen Yim, A. , Tao, Y. , Wong, F. L. , Isobe, S. , Wong, C. F. , Wong, K. S. , Xu, C. , Li, C. , Wang, Y. , Guan, R. , Sun, F. , Fan, G. , … Lam, H. M. (2014). Identification of a novel salt tolerance gene in wild soybean by whole‐genome sequencing. Nature Communications, 5, 4340. 10.1038/ncomms5340 PMC4104456 25004933

[tpg270020-bib-0033] Qiu, J. , Wang, Y. , Wu, S. , Wang, Y. Y. , Ye, C. Y. , Bai, X. , Li, Z. , Yan, C. , Wang, W. , Wang, Z. , Shu, Q. , Xie, J. , Lee, S. H. , & Fan, L. (2014). Genome re‐sequencing of semi‐wild soybean reveals a complex *Soja* population structure and deep introgression. PLoS ONE, 9, e108479. 10.1371/journal.pone.0108479 25265539 PMC4181298

[tpg270020-bib-0034] Sazonovs, A. , & Barrett, J. C. (2018). Rare‐variant studies to complement genome‐wide association studies. Annual Review of Genomics and Human Genetics, 19, 97–112. 10.1146/annurev-genom-083117-021641 29801418

[tpg270020-bib-0035] Schilthuizen, M. , Hoekstra, R. F. , & Gittenberger, E. (2001). The ‘rare allele phenomenon’ in a ribosomal spacer. Molecular Ecology, 10, 1341–1345. 10.1046/j.1365-294x.2001.01282.x 11380889

[tpg270020-bib-0036] Schmutz, J. , Cannon, S. B. , Schlueter, J. , Ma, J. , Mitros, T. , Nelson, W. , Hyten, D. L. , Song, Q. , Thelen, J. J. , Cheng, J. , Xu, D. , Hellsten, U. , May, G. D. , Yu, Y. , Sakurai, T. , Umezawa, T. , Bhattacharyya, M. K. , Sandhu, D. , Valliyodan, B. , … Jackson, S. A. (2010). Genome sequence of the palaeopolyploid soybean. Nature, 463, 178–183. 10.1038/nature08670 20075913

[tpg270020-bib-0037] Shen, Y. , Du, H. , Liu, Y. , Ni, L. , Wang, Z. , Liang, C. , & Tian, Z. (2019). Update soybean Zhonghuang 13 genome to a golden reference. Science China Life Sciences, 62, 1257–1260. 10.1007/s11427-019-9822-2 31444683

[tpg270020-bib-0038] Shen, Y. , Liu, J. , Geng, H. , Zhang, J. , Liu, Y. , Zhang, H. , Xing, S. , Du, J. , Ma, S. , & Tian, Z. (2018). De novo assembly of a Chinese soybean genome. Science China Life Sciences, 61, 871–884. 10.1007/s11427-018-9360-0 30062469

[tpg270020-bib-0039] Shimomura, M. , Kanamori, H. , Komatsu, S. , Namiki, N. , Mukai, Y. , Kurita, K. , Kamatsuki, K. , Ikawa, H. , Yano, R. , Ishimoto, M. , Kaga, A. , & Katayose, Y. (2015). The *Glycine max* cv. Enrei genome for improvement of Japanese soybean cultivars. International Journal of Genomics, 2015, 358127. 10.1155/2015/358127 26199933 PMC4493290

[tpg270020-bib-0040] Singh, G. (2010). The soybean: Botany, production and uses. CABI Publishing. 10.5860/choice.48-5085

[tpg270020-bib-0041] Song, Q. , Hyten, D. L. , Jia, G. , Quigley, C. V. , Fickus, E. W. , Nelson, R. L. , & Cregan, P. B. (2015). Fingerprinting soybean germplasm and its utility in genomic research. G3, 5, 1999–2006. 10.1534/g3.115.019000 26224783 PMC4592982

[tpg270020-bib-0042] Song, J. , Sun, S. , Ren, H. , Grison, M. , Boutté, Y. , Bai, W. , & Men, S. (2019). The SMO1 family of sterol 4α‐methyl oxidases is essential for auxin‐ and cytokinin‐regulated embryogenesis. Plant Physiology, 181, 578–594. 10.1104/pp.19.00144 31341004 PMC6776873

[tpg270020-bib-0043] The 1000 Genomes Project Consortium . (2012). An integrated map of genetic variation from 1,092 human genomes. Nature, 491, 56–65. 10.1038/nature11632 23128226 PMC3498066

[tpg270020-bib-0044] Torkamaneh, D. , Laroche, J. , Tardivel, A. , O'Donoughue, L. , Cober, E. , Rajcan, I. , & Belzile, F. (2018). Comprehensive description of genomewide nucleotide and structural variation in short‐season soya bean. Plant Biotechnology Journal, 16, 749–759. 10.1111/pbi.12825 28869792 PMC5814582

[tpg270020-bib-0045] Vaghela, B. , Vashi, R. , Rajput, K. , & Joshi, R. (2022). Plant chitinases and their role in plant defense: A comprehensive review. Enzyme and Microbial Technology, 159, 110055. 10.1016/j.enzmictec.2022.110055 35537378

[tpg270020-bib-0046] Valliyodan, B. , Brown, A. V. , Wang, J. , Patil, G. , Liu, Y. , Otyama, P. I. , Nelson, R. T. , Vuong, T. , Song, Q. , Musket, T. A. , Wagner, R. , Marri, P. , Reddy, S. , Sessions, A. , Wu, X. , Grant, D. , Bayer, P. E. , Roorkiwal, M. , Varshney, R. K. , … Nguyen, H. T. (2021). Genetic variation among 481 diverse soybean accessions, inferred from genomic re‐sequencing. Scientific Data, 8, 50. 10.1038/s41597-021-00834-w 33558550 PMC7870887

[tpg270020-bib-0047] Valliyodan, B. , Cannon, S. B. , Bayer, P. E. , Shu, S. , Brown, A. V. , Ren, L. , Jenkins, J. , Chung, C. Y. , Chan, T. F. , Daum, C. G. , Plott, C. , Hastie, A. , Baruch, K. , Barry, K. W. , Huang, W. , Patil, G. , Varshney, R. K. , Hu, H. , Batley, J. , … Nguyen, H. T. (2019). Construction and comparison of three reference‐quality genome assemblies for soybean. The Plant Journal, 100, 1066–1082. 10.1111/tpj.14500 31433882

[tpg270020-bib-0048] Valliyodan, B. , Qiu, D. , Patil, G. , Zeng, P. , Huang, J. , Dai, L. , Chen, C. , Li, Y. , Joshi, T. , Song, L. , Vuong, T. D. , Musket, T. A. , Xu, D. , Shannon, J. G. , Shifeng, C. , Liu, X. , & Nguyen, H. T. (2016). Landscape of genomic diversity and trait discovery in soybean. Scientific Reports, 6, 23598. 10.1038/srep23598 27029319 PMC4814817

[tpg270020-bib-0049] Wang, Q. , Dhindsa, R. S. , Carss, K. , Harper, A. R. , Nag, A. , Tachmazidou, I. , Vitsios, D. , Deevi, S. V. V. , Mackay, A. , Muthas, D. , Hühn, M. , Monkley, S. , Olsson, H. , Wasilewski, S. , Smith, K. R. , March, R. , Platt, A. , Haefliger, C. , & Petrovski, S. , AstraZeneca Genomics Initiative . (2021). Rare variant contribution to human disease in 281,104 UK Biobank exomes. Nature, 597(7877), 527–532. 10.1038/s41586-021-03855-y 34375979 PMC8458098

[tpg270020-bib-0050] Wang, B. , Huang, D. , Cao, C. , & Gong, Y. (2023). Insect α‐amylases and their application in pest management. Molecules, 28, 7888. 10.3390/molecules28237888 38067617 PMC10708458

[tpg270020-bib-0051] Wood, A. R. , Esko, T. , Yang, J. , Vedantam, S. , Pers, T. H. , Gustafsson, S. , Chu, A. Y. , Estrada, K. , Luan, J. , Kutalik, Z. , Amin, N. , Buchkovich, M. L. , Croteau‐Chonka, D. C. , Day, F. R. , Duan, Y. , Fall, T. , Fehrmann, R. , Ferreira, T. , Jackson, A. U. , … Frayling, T. M. (2014). Defining the role of common variation in the genomic and biological architecture of adult human height. Nature Genetics, 46, 1173–1186. 10.1038/ng.3097 25282103 PMC4250049

[tpg270020-bib-0052] Xie, M. , Chung, C. Y. , Li, M. W. , Wong, F. L. , Wang, X. , Liu, A. , Wang, Z. , Leung, A. K. , Wong, T. H. , Tong, S. W. , Xiao, Z. , Fan, K. , Ng, M. S. , Qi, X. , Yang, L. , Deng, T. , He, L. , Chen, L. , Fu, A. , … Lam, H. M. (2019). A reference‐grade wild soybean genome. Nature Communications, 10, 1216. 10.1038/s41467-019-09142-9 PMC6418295 30872580

[tpg270020-bib-0053] Zhang, C. , Huang, L. , Zhang, H. , Hao, Q. , Lyu, B. , Wang, M. , Epstein, L. , Liu, M. , Kou, C. , Qi, J. , Chen, F. , Li, M. , Gao, G. , Ni, F. , Zhang, L. , Hao, M. , Wang, J. , Chen, X. , Luo, M. C. , … Fu, D. (2019). An ancestral NB‐LRR with duplicated 3′ UTRs confers stripe rust resistance in wheat and barley. Nature Communications, 10, 4023. 10.1038/s41467-019-11872-9 PMC6731223 31492844

[tpg270020-bib-0054] Zhang, H. , Jiang, H. , Hu, Z. , Song, Q. , & An, Y. C. (2022). Development of a versatile resource for post‐genomic research through consolidating and characterizing 1500 diverse wild and cultivated soybean genomes. BMC Genomics, 23, 250. 10.1186/s12864-022-08326-w 35361112 PMC8973893

[tpg270020-bib-0055] Zhou, Z. , Jiang, Y. , Wang, Z. , Gou, Z. , Lyu, J. , Li, W. , Yu, Y. , Shu, L. , Zhao, Y. , Ma, Y. , Fang, C. , Shen, Y. , Liu, T. , Li, C. , Li, Q. , Wu, M. , Wang, M. , Wu, Y. , Dong, Y. , … Tian, Z. (2015). Resequencing 302 wild and cultivated accessions identifies genes related to domestication and improvement in soybean. Nature Biotechnology, 33, 408–414. 10.1038/nbt.3096 25643055

