# Supplementary material for: Landscape of rare‐allele variants in cultivated and wild soybean genomes
Source: Plant Genome. 2025 Mar 27;18(2):e70020. doi: 10.1002/tpg2.70020 (PMC11949740; doi:10.1002/tpg2.70020)
Supplement: Supplementary file 6 — Figure S1. Allele frequencies of the variants at Chr05_7237778 in G. soja and G. max populations. Blue represents the soybean accessions with the “A” allele, orange represents the accessions with the “G” allele. [file TPG2-18-e70020-s006.docx]

A G

44.5%

55.5%

0.6%

99.4%

G. soja

(*n* = 182)

G. max

(*n* = 1287)

**Figure S1. Allele frequencies of the variants at Chr05_7237778 in *G. soja* and *G. max* populations.** Blue represents the soybean accessions with the “A” allele, orange represents the accessions with the “G” allele.
